# Cu_2_ZnSnS_4_ absorption layers with controlled phase purity

**DOI:** 10.1038/srep09291

**Published:** 2015-03-24

**Authors:** Chia-Ying Su, Chiu -Yen Chiu, Jyh-Ming Ting

**Affiliations:** 1 Department of Materials Science and Engineering, National Cheng Kung University, Tainan, Taiwan; 2 Material and Chemical Research, Industrial Technology Research Institute, Hsinchu, Taiwan; 3 Research Center for Energy Technology and Strategy, National Cheng Kung University

## Abstract

We report the synthesis and characterization of Cu_2_ZnSnS_4_ (CZTS) with controlled phase purity. The precursor was first prepared using sequential electrodeposition of Cu, Zn, and Sn in different orders. The Cu/(Sn+Zn) ratio in each stacking order was also varied. The precursor was subjected to annealing at 200°C and sulfurization at 500°C in a 5%-H_2_S/Ar atmosphere for the formation of CZTS. The phase evolutions during the electrodeposition and annealing stages, and the final phase formation at the sulfurization stage were examined using both x-ray diffractometry and Raman spectroscopy, both of which are shown to be complimentary tools for phase identification. Detailed growth path is therefore reported. We also demonstrate by controlling the stacking order and the Cu/(Sn+Zn) ratio, CZTS with a phase purity as high as 93% is obtained.

Electrical energy generated from various thin film solar cells are of great importance due to the depleting natural resources in the earth. Among these thin film solar cells, amorphous silicon (α-Si), cadmium telluride (CdTe), and copper indium gallium selenium (CIGS) are the major non-organic solar cells^1^. CIGS thin film solar cell is attractive due to the fabrication cost and conversion efficiency (~20%)^2^,^3^. However, a potential drawback of CIGS thin film solar cell is that the absorber layer contains rare earth elements of In and Ga, both of which are likely to be in shortage in the future. Therefore, alternatives are being intensively sought. As a result, semiconductor materials, quaternary Cu_2_ZnSnS_4_ (CZTS) and Cu_2_ZnSnSe_4_ (CZTSe) compounds are receiving increasing attentions^4^. The Zn and Sn replace the In to form a chalcopyrite-like structure^5^. CZTS exhibits a high absorption coefficient (>10^4^ cm^−1^) and an energy gap (~1.5 eV) matching the visible light spectrum^6^. The energy band gap can also be modified through varying the phase purity and composition^7^,^8^. The vacuum processes used for the fabrication of CZTS absorber layers are similar to that of the CIGS and include sputter and evaporative deposition techniques. In one type of the evaporative processes, elemental Cu, Sn, and S, and binary ZnS sources are co-evaporated onto the substrates^4^. In another type, various elements (Cu, Sn, and Zn) and compounds (ZnS and SnS_2_) are evaporated in sequences^9^,^10^,^11^. In sputter deposition techniques, both co-sputter and sequential sputter deposition have been used. There are also several non-vacuum processes reported. The first report on CZTS thin film solar cell fabricated under non- vacuum condition is a sol-gel process^12^. The obtained cell has a conversion efficiency of 1.01%. CZTS thin films were also deposited using spray pyrolysis of an aqueous solution containing cupric chloride, zinc acetate, stannic chloride, and thiourea onto substrates heated to a temperature between 643–683 K^5^. However, an amorphous ZnS layer was observed on the surface of the CZTS film and led to increased electrical resistance. ZnS as well as was found at the interface between CZTS and Mo substrate which contributes to the high short-circuit current density, high series resistance found in the resulting cells^13^. CZTS/CZTSe thin films can also be prepared from the sintering of nanoparticles, which can be made, for example, using high-temperature arrested precipitation at 280°C^14^ and hot injection^15^.

Another non-vacuum alternative is electroplating, which is a scalable process. Electroplating is a room-temperature process. Also, this technique eliminates the problem of residual carbon that is often arisen in other non-vacuum processes^16^,^17^,^18^. CZTS thin films can be obtained using co-plating or sequential plating of the constituent elements with^19^,^20^ or without S^21^,^22^,^23^,^24^. Post-plating sulfurization is required for electroplated film with no sulfur. Compared to the sequential plating technique, the co-plating technique requires a longer deposition time^19^,^22^ and more organic additives^22^,^25^, and is difficult in maintaining the desired characteristics of the electrolyte^19^. In sequential electroplating, almost all of the CZTS absorber layers were electroplated on Mo-coated substrates with a stacking order of Cu/Sn/Zn without Ref. 25 or with Ref. 26 a Pd layer on the Mo surfaces. Recently, Cu/Zn/Sn precursor layer was prepared using a sequential plating technique^27^. The resulting CZTS cell is exhibiting the highest conversion efficiency (7.3%) among all the CZTS cells whose absorber layers are prepared using electroplating techniques^28^. This indicates that the stacking order play an important role in the performance of the cell. However, only very limited studies, in which vacuum processes were used, have shown that the stacking order affects the morphology, composition, and phase of the resulting CZTS layer and therefore the cell performance. For example, six different stacking orders of Cu, Sn, and Zn were electron-beam evaporated onto Mo/glass substrates to create 3-layered films^29^. It was found that the resulting morphologies and compositions were different in different stacking orders, and a higher conversion efficiency can be obtained by having Cu and Sn adjacent to each other. Different stacking orders of Cu, Zn and Sn were sputter deposited to create multi-layered films^30^. It was found that some of the multi-layered films did not transform into “good quality CZTS films” after the sulfurization. Voids were observed in some of the all samples.

Regardless of the process methods, an important issue of concern is the phase purity. It is known that impurity phases lead to high series resistance and hence limit the conversion efficiency^21^,^51^,^52^,^57^. Surface impurity Cu_X_S can be removed by KCN solution. However, the bulk impurity such as Cu_2_SnS_3_ ZnS, and Sn_x_S_y_ co-exist with the Cu_2_ZnSnS_4_ phase^21^,^25^,^52^,^53^,^54^ and cannot be removed by post-synthesis treatment. Despite of the recognition of the disadvantages of impurity phases, there is no report showing quantitative data^55^,^56^,^58^,^59^. In this study, we have prepared multi-layered precursor films having different stacking orders of Cu, Zn, and Sn. Also, in each stacking order, three different Cu/(Sn+Zn) ratios were studied. Each layer was electroplated sequentially. The obtained precursor films were then annealed and sulfurized in a closed quartz tube at elevated temperatures. Effects of the stacking order and the Cu/(Sn+Zn) ratio on the characteristics of the resulting CZTS are presented and discussed.

## Results

**Figure 1:**
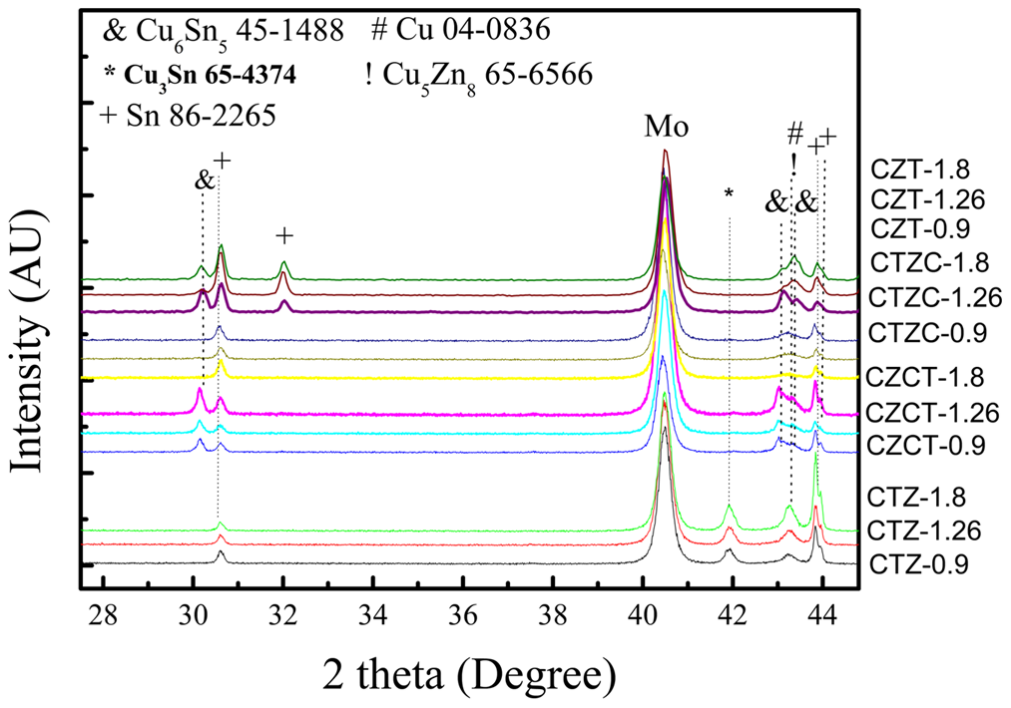
XRD patterns of as-plated films. The numbers follows the sample IDs represent the Cu/(Sn+Zn) ratios.

**Figure 2:**
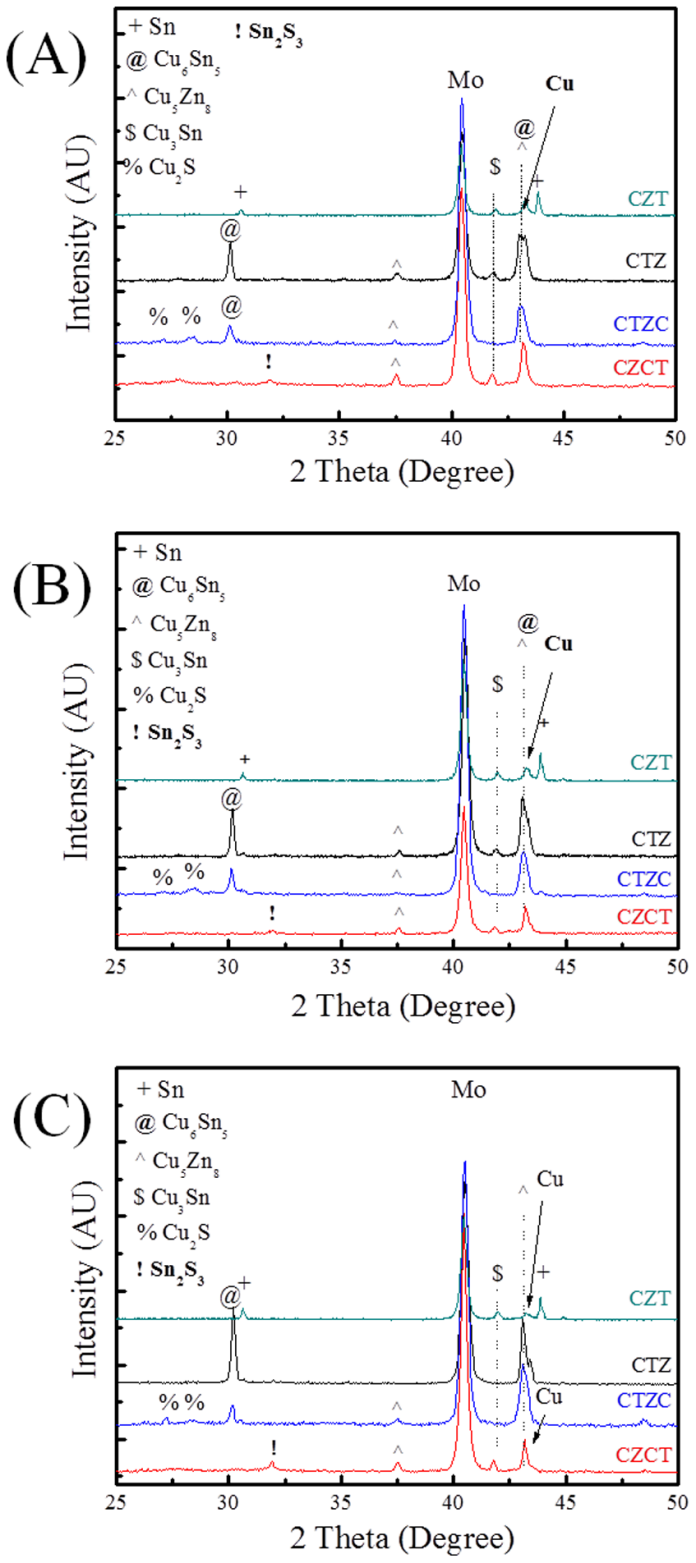
XRD patterns for heat treated samples. (A) Cu/(Sn+Zn) = 1.8, (B) Cu/(Sn+Zn) = 1.26, and (C) Cu/(Sn+Zn) = 0.9. The heat treatment was performed at 200°C for 30 min in H_2_S.

**Figure 3:**
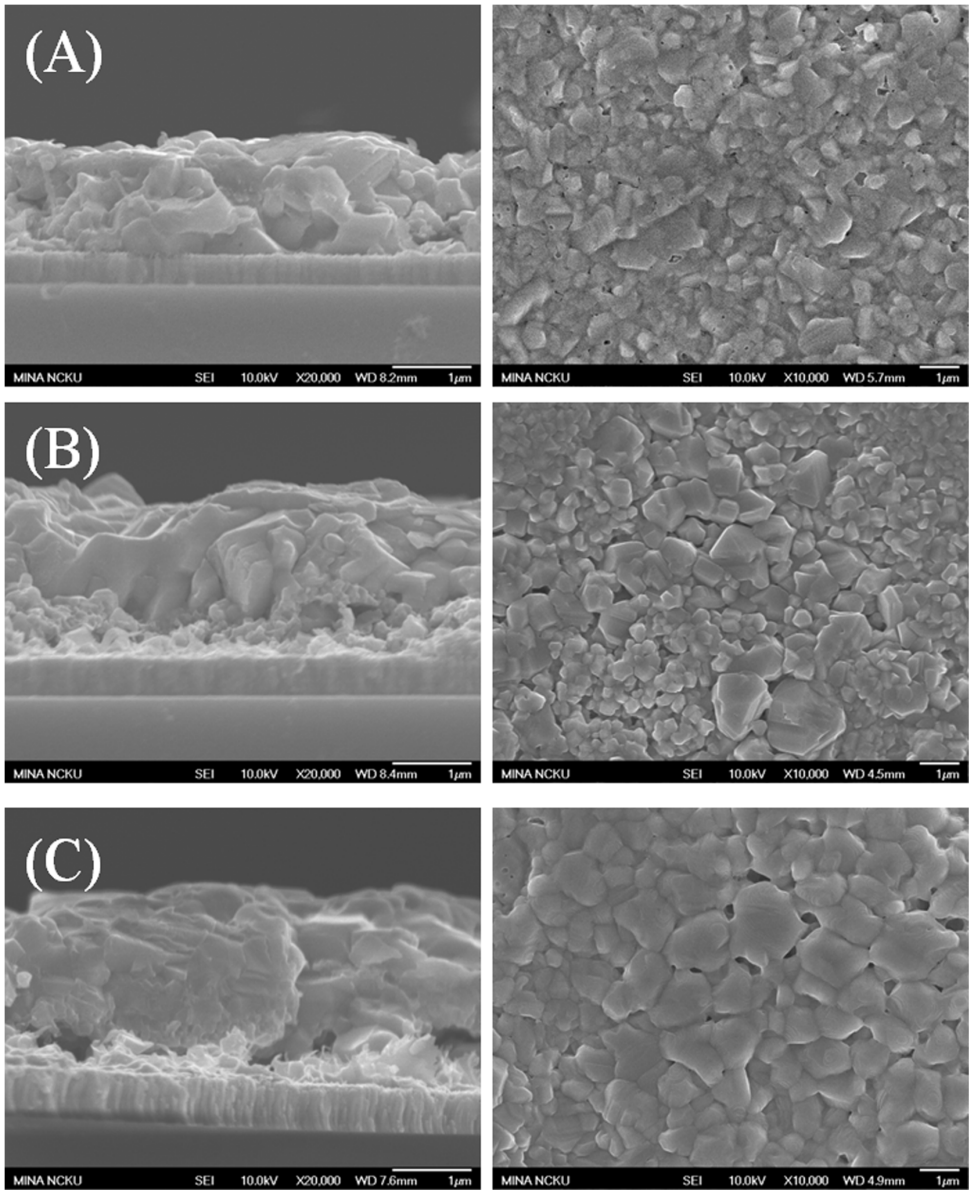
SEM cross-sectional and top view of sulfuirzed CZTS samples: (A) CTZ-0.9, (B) CTZ-1.26, and (C) CTZ-1.8.

**Figure 4:**
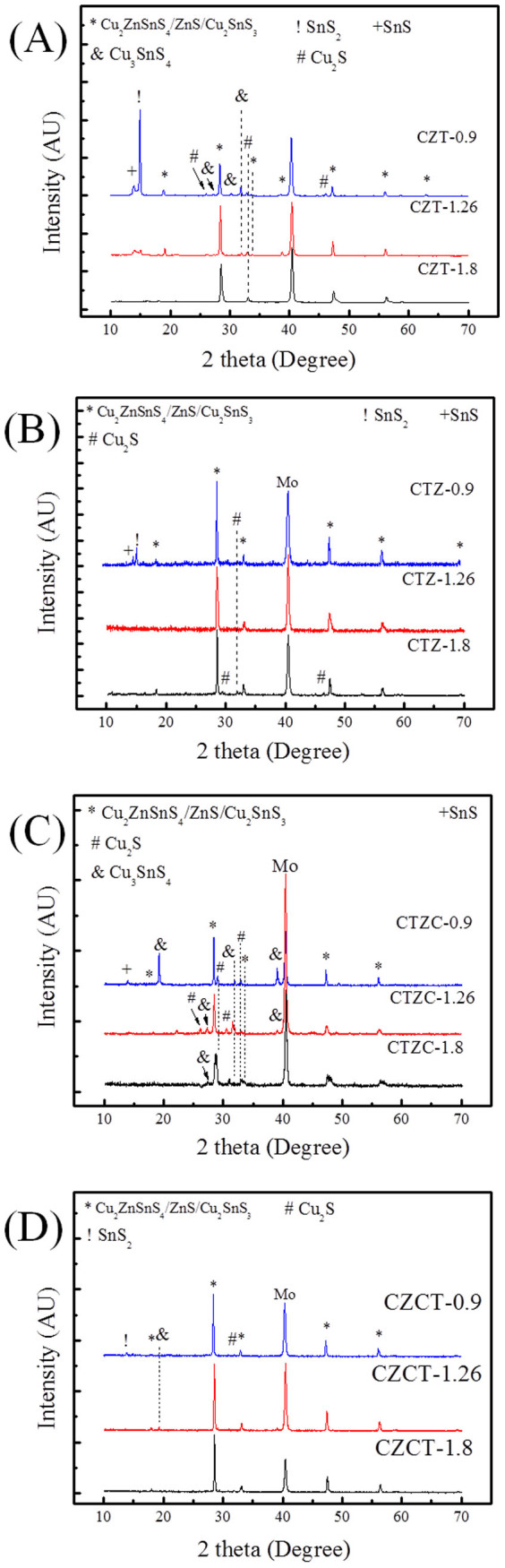
XRD patterns of CZTS films. (A) CZT, (B) CTZ, (C) CTZC, and (D) CZCT.

**Figure 5:**
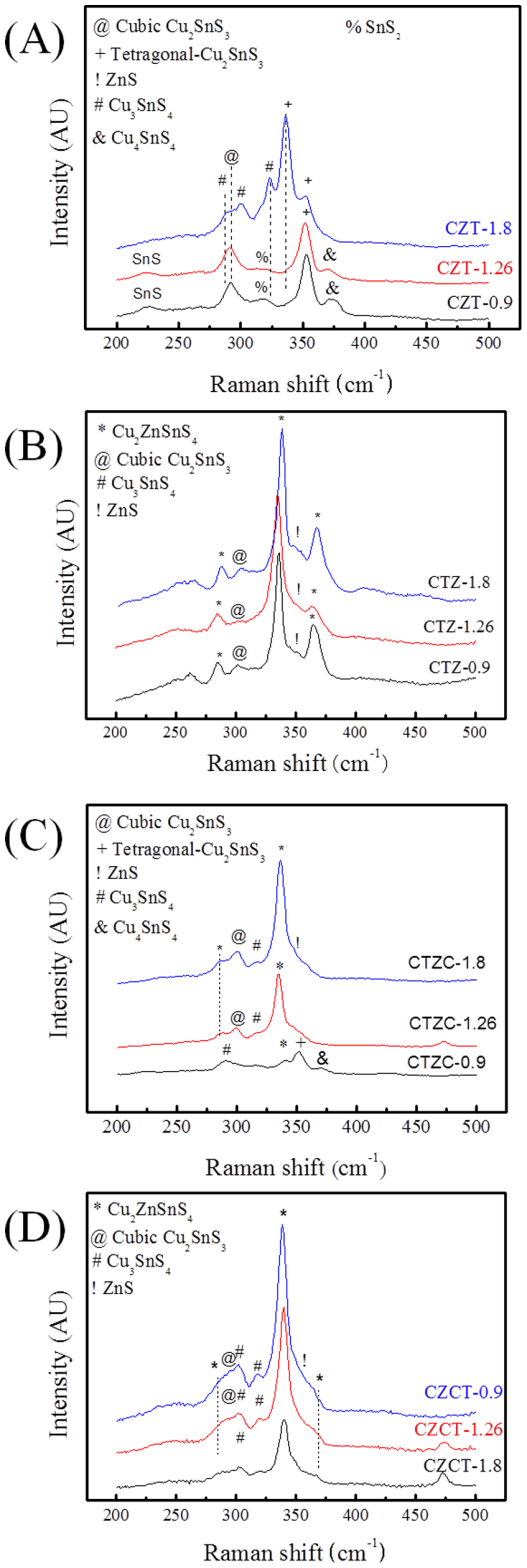
Raman spectra of CZTS films. (A) CZT, (B) CTZ, (C) CTZC, and (D) CZCT.

**Figure 6:**
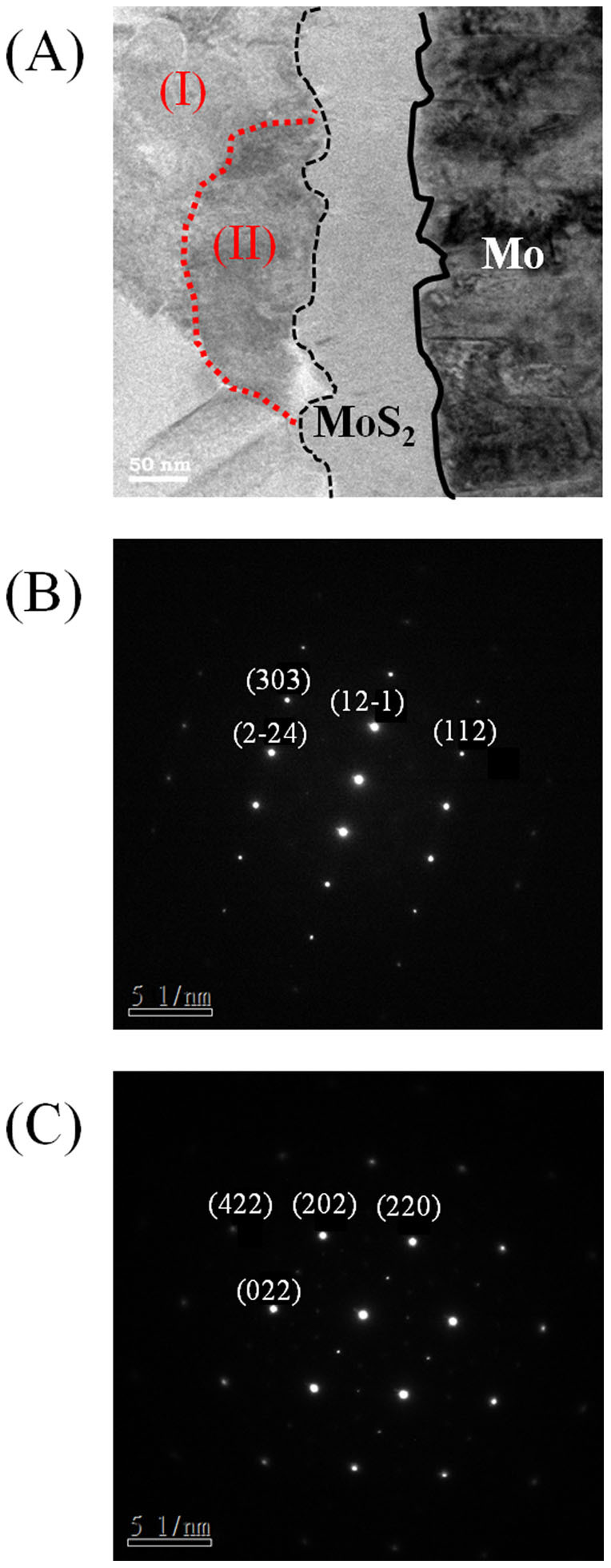
TEM analysis of sulfurized CTZ-1.8sample. (A) Bright field image. (B) Diffraction patterns of areas (B) I and (C) II.

XRD analysis (Fig. 1) shows that the as-plated CZT sample consists of Cu_6_Sn_5_, Cu, and Sn phases, as-plated CTZ sample consists of Cu_5_Zn_8_, Cu_3_Sn, Cu, and Sn phases, as-plated CTZC sample consists of Cu_5_Zn_8_, Cu, and Sn phases, and as-plated CZCT sample films consists of Cu_5_Zn_8_, Cu_6_Sn_5_, Cu, and Sn phases. These phases are either two-element compounds or pure elements. For clarity, Table 1 summarizes the phases that were observed in different cases. As shown in the table, only Cu-Sn and/or Cu-Zn compounds were found. Also, Cu_5_Zn_8_ exists in all the as-plated samples, except the as-plated CZT as shown in Table 1. Furthermore, there is no elemental Zn in the as-plated CZT. The as-plated samples were then subjected to annealing at 200°C for 30 min in H_2_S. Figs. 2A to 2C show the XRD patterns of the annealed samples. After the annealing, the obtained phases include Cu_5_Zn_8_, Cu_6_Sn_5_, Cu_3_Sn, Cu, Sn, Cu_2_S and Sn_2_S_3_. The results are also summarized in Table 1. In the annealed CZT, the Cu_6_Sn_5_ phase in the as-plated state disappears and Cu_3_Sn forms after the annealing. After annealing, a new phase, i.e., Cu_6_Sn_5_, forms and Sn disappears in the CTZ sample. For CTZC, the annealing leads to the formation of additional phases of Cu_6_Sn_5_ and Cu_2_S. For Sample CZCT, Cu_6_Sn_5_ disappears while two additional phases of Cu_3_Sn and Sn_2_S_3_ form after the annealing. To obtain CZTS, the annealed samples were then sulfurized. After being subjected to KCN solution treatment, the sulfurized samples were analyzed. There is no obvious relation between the densification and the stacking order after the sulfurization. However, it was found that the higher the Cu/(Sn+Zn) ratio the more porous the sulfurized film as shown in Fig. 3 for selected sulfurized CTZ samples. While the phase composition of the annealed sample depends only on the stacking order but not the Cu/(Sn+Zn) ratio, the phase composition of the sulfurized sample varies with not only the stacking order but also the Cu/(Sn+Zn) ratio. Fig. 4 shows the XRD patterns of sulfurized samples. All the samples have the commonly observed surface Cu_2_S^21^,^31^, which can be removed after the KCN treatment. In the sulfurized CZT, the XRD patterns show that only CZT-0.9 and -1.26 but not CZT-1.8 have Cu_3_SnS_4_, SnS, and SnS_2_. On the other hand, all three samples show diffractions peaks that belong to Cu_2_ZnSnS_4_, ZnS, and/or Cu_2_SnS_3_. These three phases have nearly overlapping diffraction peaks that cannot be easily indentified. However, these peaks surely belong to the kesterite structure of CZTS^32^. Although XRD is a common tool for the determination of crystalline phase, there are situations that the diffraction peaks of different phases nearly overlap, as mentioned above. Furthermore, some minor phases often cannot be detected by XRD. Therefore Raman analysis is used as a supplementary tool as reported earlier^31^,^33^,^34^. Fig. 5 gives the Raman spectra of the sulfurized samples. As mentioned above, the XRD patterns show that CZT-1.8 does not contain Cu_3_SnS_4_. However, a minor amount of Cu_3_SnS_4_ was detected by the Raman analysis. Also, Cu_4_SnS_4_ was not detected by the XRD but found during the Raman analysis for Samples CZT-0.9 and -1.26. Furthermore, although XRD cannot clearly reveal the true phase(s) of the aforementioned overlapping peaks, the Raman analysis indicates that CZT-0.9 contains only tetragonal Cu_2_SnS_3_ and CZT-1.26 and -1.8 contain both cubic and tetragonal Cu_2_SnS_3_. By the same approach, i.e., using both XRD and Raman for the phase analysis, Table 2 summarizes all the phases detected by XRD and Raman. It is apparent that some of the phases that cannot be found or identified by the XRD analysis can be realized using the Raman analysis. The phase percentage of CZTS given in Table 2 was determined from the peak area in the Raman spectra. There is no CZTS phase in sulfurized CZT. The amount of CZTS in other samples varies. It is seen that a higher Cu/(Sn+Zn) gives a higher CZTS percentage. Considering samples having different stacking orders, the CTZ group has the highest CZTS percentages, followed by the CZCT group and then the CTZC group. Furthermore, it is seen in the sulfurized CTZ-1.8, i.e., the most complete sulfurization film, there is basically no Cu_2_SnS_3_ (<0.4%) and only 6.1% of ZnS. TEM analysis supports this result since only CZTS and ZnS were observed as shown in Fig. 6A. A CZTS and ZnS grains are labeled as I and II, respectively. Their pertinent diffraction patterns are shown in Figs. 6B and 6C, respectively. Area I is a Cu_2_ZnSnS_4_ single crystal with a zone axis of [−1 1 1]. Area II is a ZnS single crystal with a zone axis of [−1 1 −1]. No cubic-Cu_2_SnS_3_ phase is seen which echoes the Raman analysis.

## Discussion

**Figure 7:**
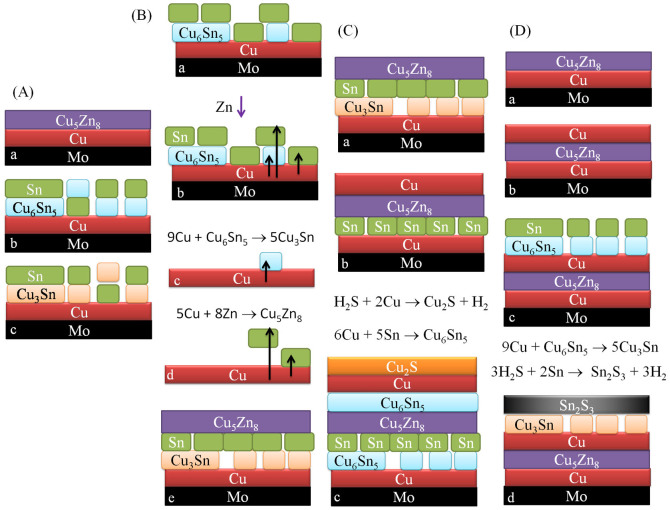
Phase formation during each stage for Samples (A) CZT, (B) CTZ, (C) CTZC, and (D) CZCT.

From the above result, phase formation during each stage is schematically presently in Fig. 7. As summarized in Table 1, there is no Zn-Sn alloy or compound but only Cu-Sn and/or Cu-Zn compounds. In room temperature electrodeposited Cu-Zn samples, phases ranging from the entire spread of the binary phase diagram, including β-CuZn, γ-Cu_5_Zn_8_ and ε-CuZn_5_, have been observed^35^,^36^,^37^. Among them, Cu_5_Zn_8_ is a stable room temperature phase^38^. Therefore, Cu_5_Zn_8_ exists in all the as-plated samples, except the as-plated CZT, which has no element Zn either. It was found that after the deposition of Cu and Zn in sequence, only Cu_5_Zn_8_ film was found on top of the Cu as illustrated in Fig. 7Aa and shown in Fig. S1 in the Supporting Information, indicating all the Zn reacted with the Cu. The Cu_5_Zn_8_ layer, however, disappeared after the subsequent pulsed plating of Sn. This is attributed to the much higher electrical resistance of Cu_5_Zn_8_, as compared to Cu (2 orders of magnitude higher). The high electrical resistance would create a sudden voltage drop that cracks the brittle Cu_5_Zn_8_ layer. In the meantime, the plated Sn reacted with the exposed Cu to form hexagonal η-Cu_6_Sn_5_ phase^39^, leaving Sn, Cu_6_Sn_5_, and Cu in the sample as shown in Fig. 7Ab and Table 1. The co-existence of elemental β-Sn (tetragonal) and Cu (hexagonal), and Cu_6_Sn_5_ is commonly observed in electroplate Cu-Sn along with^40^. In as-plated CTZ, no Cu_6_Sn_5_ but Cu_3_Sn, Cu_5_Zn_8_, Cu, and Sn were observed. Thermodynamically, Cu_6_Sn_5_ is more stable than Cu_3_Sn^41^,^42^. In CTZ, after Cu and Sn were first deposited in sequence (Fig. 2Bb), the stable Cu_6_Sn_5_ phase was indeed identified by XRD analysis, as shown in Fig. S2 in the Supporting Information. This is also illustrated in Fig. 7Ba. Subsequent deposition of Zn then leads to not only the formation of the stable Cu_5_Zn_8_ phase but also the transformation of Cu_6_Sn_5_ to Cu_3_Sn. A possible mechanism is given below and also shown schematically in Fig. 7Bb and Bc. First of all, we believe that the diffusion of Cu toward to the surface occurs during the Zn plating (Fig. 7Bb). Although such diffusion is normally induced by thermal energy^43^, the pulsed power during the Zn plating could provide a driving force for the Cu diffusion. Also, the strong affinity between Cu and Zn can contribute to the diffusion. The Cu atoms that diffuse into the Cu_6_Sn_5_ result in excess Cu in the phase. As a result, the Cu_6_Sn_5_ is transformed into Cu_3_Sn, as shown in Fig. 7Bc. The Cu atoms that diffuse through the Sn react with the depositing Zn to form Cu_5_Zn_8_, as shown in Fig. 7Bd. It is noted that the reaction between Cu and Zn is much favorable thermodynamically than that between Cu and Sn^42^,^44^. This is also analogous to the case of soldering, which reports that during the soldering of Sn-Zn-Ag, Sn-Zn-Ag-Al-Ga, or Sn-Bi-In-Zn onto Cu substrate, Cu diffuses into the solder and preferentially reacts with Zn to form Cu_5_Zn_8_^45^,^46^. As-plated CTZ therefore consists of Cu, Sn, Cu_5_Zn_8_, and Cu_3_Sn (Table 1 and Fig. 7Be). In as-plated CTZ, a less stable Cu-Sn phase, i.e., Cu_3_Sn, was found as mentioned above. This structure is the same as that of the as-plated CTZC before the top layer Cu plating as shown in Fig. 7Ca. After the top Cu layer plating the Cu_3_Sn disappears also without the formation of any other Cu-Sn phase, as shown in Fig. 7Cb. For the as-plated CZCT, the initial plating of Cu and Zn in sequence leads to the formation of stable Cu_5_Zn_8_ on the remaining Cu (Fig. 7Da). Subsequent plating of additional Cu does not change the phases as shown in Fig. 7Db. Final plating of Sn then allows the reaction between the Sn and the Cu to form stable Cu_6_Sn_5_. As a result, as-plated CZCT consists of Cu_5_Zn_8_, Cu_6_Sn_5_, Cu, and Sn, as shown in Fig. 7Dc).

Now we discuss the annealed samples in which XRD analysis shows the existence of Cu_5_Zn_8_, Cu_6_Sn_5_, Cu_3_Sn, Cu, Sn, Cu_2_S and Sn_2_S_3_ (Table 1). The annealing of CZT leads to the disappearance of the Cu_6_Sn_5_. The only reason that this happened is that more Cu atoms diffuse into Cu_6_Sn_5_ during the annealing to form Cu_3_Sn^47^, as shown in Fig. 7Ac, following the mechanism that is described above during the plating. For the as-plated CTZ, Cu_6_Sn_5_ forms after the annealing. It is believed that Cu diffuses upwards to react with Sn to form Cu_6_Sn_5_ (Fig. 7Be) which is thermodynamically favorable^41^. The formation of Cu_6_Sn_5_ is also contributed by the reaction between Sn and Cu_3_Sn^48^,^49^, as also shown in Fig. 7Be. Both reactions, as shown in Fig. 7Be, result in the disappearance of Sn. For the as-plated CTZC (Fig. 7Cb), the annealing let the bottom Cu and Sn react to form Cu_6_Sn_5_, while the top Cu react with H_2_S to form Cu_2_S, as shown in Fig, 7Cc. For the as-annealed CZCT, two additional phases of Cu_3_Sn and Sn_2_S_3_ were observed. As mentioned above, Cu_3_Sn can be obtained through the diffusion of Cu into Cu_6_Sn_5_ during the annealing which also results in the disappearance of Cu_6_Sn_5_, as shown in Fig. 7Dd. Furthermore Sn reacts with H_2_S to form Sn_2_S_3_ which also leads to the disappearance of Sn, as also shown in Fig. 7Dd.

The annealed samples were subsequently sulfurized to obtain CZTS. As mentioned above, no obvious relation between the densification and the stacking order after the sulfurization was observed and a higher Cu/(Sn+Zn) ratio gives a more porous sulfurized film. This is attributed to the fact that Cu reacts with sulfur easily^31^. A higher Cu/(Sn+Zn) ratio leads to more Cu/S reaction which involves the diffusion of Cu to the surface for the formation of Cu_x_S (Cu_2_S or CuS), leaving pores in the film. Also, after the surface Cu_x_S is removed by KCN, the pores are formed too. As a result, the higher the Cu/(Sn+Zn) ratio the more porous the sulfurized film. On the other hand, the phase composition of the sulfurized sample varies with both the stacking order and Cu/(Sn+Zn) ratio. This is different from the annealed samples whose phase composition is independent of the Cu/(Sn+Zn) ratio. As mentioned above, the phase analysis was examined using both XRD and Raman and the results are shown in Table 2. It is understood that the formation of the phases in the sulfurized samples is due to the complicated interplays among many solid-state and solid-gas reactions. It is not the intention of this paper to discuss the detailed formation mechanism or the growth path. We will address here the appearance of CZTS in the sulfurized samples. From Table 2, it is seen that no CZTS phase in sulfurized CZT as there is no Zn in its as-annealed state (Table 1), while the amount of CZTS in other samples varies. Firstly, we consider the effect of Cu/(Sn+Zn) ratio, i.e., a higher Cu/(Sn+Zn) gives a higher CZTS percentage. Considering both Cu-rich and Cu-poor samples, the last stage to form CZTS is through Cu_2_SnS_3_ + ZnS → Cu_2_ZnSnS_4_^23^. The formation of Cu_2_SnS_3_ involves reaction of Cu_2-x_S with SnS_2_. In this study, a higher Cu/(Sn+Zn) ratio was obtained by increasing the Cu content. Therefore, a higher Cu/(Sn+Zn) ratio results in a higher percentage of Cu_2_S, as shown in Fig. S3 in the Supporting Information. As a result, more Cu_2_SnS_3_ and hence more Cu_2_ZnSnS_4_ is obtained at a higher Cu/(Sn+Zn) ratio. Then we consider the effect of stacking orders. Comparing different stacking orders, the CTZ has the highest CZTS percentage, followed by the CZCT and then the CZT. This can be realized by also considering the last stage reaction to form CZTS shown above^23^,^50^. When there is no concurrent reduction of both cubic Cu_2_SnS_3_ and ZnS, the sulfurization is less complete, e.g., CZCT groups. For the CTCZ group, the increasing CZTS phase is accompanied by decreasing ZnS but increasing cubic Cu_2_SnS_3_. This indicates that the above reaction is hindered such that the CZTS phase is the lowest in the CTZC group. The most complete sulfurization group is the CTZ group where a higher percentage of CZTS is clearly accompanied by reduced percentages of both cubic Cu_2_SnS_3_ and ZnS in the sample. As a result, by varying the Cu/(Sn+Zn) ratio and the stacking order, the most completely sulfurized film, i.e, CTZ-1.8, has been obtained. This sample has basically no Cu_2_SnS_3_ (<0.4%) and only 6.1% of ZnS and its high phase purity has also been supported by TEM analysis.

## Conclusions

In this study, we have investigated the formation of high phase purity CZTS through examining the effect of precursor characteristics on the phase evolutions. The precursor was prepared using sequential electrodeposition of individual layers of Cu, Sn, and Zn. Three different deposition orders were studied and in each stacking order, three Cu/(Sn+Zn) ratios were used. The electrodeposited precursor was then annealed and sulfurized in sequence in a 5%-H_2_S/Ar atmosphere. It was found that the stacking order but not the Cu/(Sn+Zn) ratio affects the phase formation during the electrodeposition and annealing. However, both the stacking order and the Cu/(Sn+Zn) ratio affect the phase formation during the sulfurization. A higher Cu/(Sn+Zn) gives a higher CZTS percentage in the sulfurized sample. The effect of stacking order on the formation of CZTS phase is discussed by considering the reaction sequence. We show that the existence of and reaction among Cu_2_S, Cu_2_SnS_3_, and ZnS determine the percentage of CZTS. Furthermore, the existence of and reaction among Cu_2_S, Cu_2_SnS_3_, and ZnS depend on the stacking order. Detailed growth path is therefore reported. We also demonstrate that by controlling the stacking order and the Cu/(Sn+Zn) ratio, CZTS with a phase purity as high as 93% can be obtained.

## Methods

Copper, tin, and zinc layers were deposited on Mo-coated sodium-lime glass substrates in different sequences using an electroplating method. The electrolytes used for the plating of Cu, Zn, and Sn were 0.2 mol/L Cu_2_P_2_O_7_ + 1.06 mol/L K_4_P_2_O_7_.H_2_O + 9 × 10^−5^ mol/L SeO_2_, 0.1 mol ZnSO_4_ + 0.13 mol Na_2_SO_4_ + 0.06 mol H_3_BO_3_, and 0.1 mol SnCl_2_ + 1.2 × 10^−3^ mol CuCl_2_ + 0.16 mol C_6_H_17_N_3_O_7_, respectively. Four stacking orders were prepared for the precursors, including Cu/Zn/Sn (CZT), Cu/Sn/Zn (CTZ), Cu/Sn/Zn/Cu (CTZC), and Cu/Zn/Cu/Sn (CZCT).The total thickness of the metal precursor was 700 nm. In each type of precursor, there were three Cu/(Sn+Zn) ratios used, 0.9, 1.26, and 1.8. A high Zn/Sn atomic ratio of 1.5 was used for all the cases. A higher Zn/Sn ratio was reported to promote the formation of CZTS during sulfurization^15^. As-deposited samples are first annealed before sulfurization. Before the annealing, the quartz tube was evacuated and then back filled with 5%-H_2_S/Ar until the pressure reached 400 torr. The quartz tube was then heated to 200°C for annealing. After 30-min of annealing, the heating was then continued to 500°C for sulfurization. The sulfurization time and pressure were 60 min 560 torr, respectively. The phase composition and the crystal structure of the obtained films were determined using X-ray diffraction (XRD) and Raman spectroscopy with a 100 mW He–Ne laser (wavelength 633 nm). The morphology of was examined using field emission scanning electron microscopy (FESEM). The microstructure was also examined using transmission electron microscopy (TEM).

## Author Contributions

C.Y.S. contributed to the novelty and planning of this research, and solely conducted the experiments, characterizations, and analysis of the resulting data under the guidance J.M.T.. J.M.T. prepared figure 7 and C.Y.S. prepared the other figures. C.Y.C. provided advises of and participated in the electroplating. All authors reviewed this manuscript.

## Supplementary Material

Supplementary Information Supporting Information

## Figures and Tables

**Table 1:** XRD results of as-plated and annealing samples

	Cu_5_Zn_8_	Cu_6_Sn_5_	Cu_3_Sn	Cu	Zn	Sn	Cu_2_S	Sn_2_S_3_
As-plated CZT		X		X		X		
Annealed CZT			X	X		X		
As-plated CTZ	X		X	X		X		
Annealed CTZ	X	X	X	X				
As-plated CTZC	X			X		X		
Annealed CTCZ	X	X		X		X	X	
As-plated CZCT	X	X		X		X		
Annealed CZCT	X		X	X				X

**Table 2:** Phases identified using XRD and Raman analyses.

	Cu_2_ZnSnS_4_	ZnS	Cubic- Cu_2_SnS_3_	Tetragonal -Cu_2_SnS_3_	Cu_3_SnS_4_	Cu_4_SnS_4_	SnS_2_	SnS
CZT-0.9				45.76%	32.58%	8.64%	8.41%	4.61%
XRD	Overlapping. Determined by Raman				Y	N	Y	Y
**CZT-1.26**			**6.60%**	**48.20%**	**30.30%**	**4.20%**	2.20%	8.50%
XRD	Overlapping. Determined by Raman				Y	N	Y	Y
**CZT-1.8**			**30.00%**	**66.20%**	**3.80%**	**0.00%**	0.00%	0.00%
XRD	Overlapping. Determined by Raman				N	N	N	N
**CTZ-0.9**	**78.64%**	**15.35%**	**2.69%**	**0.00%**	**0.00%**	**0.00%**	1.55%	1.78%
XRD	Overlapping. Determined by Raman				N	N	Y	Y
**CTZ-1.26**	**89.46%**	**9.90%**	**0.64%**	**0.00%**	**0.00%**	**0.00%**	0.00%	0.00%
XRD	Overlapping. Determined by Raman				N	N	N	N
**CTZ-1.8**	**93.52%**	**6.13%**	**0.36%**	**0.00%**	**0.00%**	**0.00%**	**0.00%**	**0.00%**
XRD	Overlapping. Determined by Raman				N	N	N	N
**CTZC-0.9**	**41.80%**	**16.30%**	**4.40%**	**0.00%**	**36.80%**	**0.00%**	0.00%	0.70%
XRD	Overlapping. Determined by Raman				Y	N	N	Y
**CTZC-1.26**	**46.60%**	**11.50%**	**14.40%**	**0.00%**	**27.30%**	**0.00%**	0.00%	0.20%
XRD	Overlapping. Determined by Raman				Y	N	N	Y
**CTZC-1.8**	**70.30%**	**3.20%**	**19.50%**	**0.00%**	**7.00%**	0.00%	0.00%	0.00%
XRD	Overlapping. Determined by Raman				Y	N	N	N
**CZCT-0.9**	**78.70%**	**4.30%**	**6.60%**	**0.00%**	**8.80%**	**0.00%**	1.60%	0.00%
XRD	Overlapping. Determined by Raman				N	N	Y	N
**CZCT-1.26**	**87.00%**	**6.50%**	**3.40%**	**0.00%**	**3.10%**	**0.00%**	0.00%	0.00%
XRD	Overlapping. Determined by Raman				N	Y	N	N
CZCT-1.8	88.10%	5.90%	3.70%	**0.00%**	2.40%	0.00%	0.00%	0.00%
XRD	Overlapping. Determined by Raman				N	N	N	N
