# Supplementary material for: Cu2ZnSnS4 absorption layers with controlled phase purity
Source: Sci Rep. 2015 Mar 24;5:9291. doi: 10.1038/srep09291 (PMC4371055; doi:10.1038/srep09291)
Supplement: Supplementary Information — Supporting Information [file srep09291-s1.docx]

Cu_2_ZnSnS_4_ absorption layers with controlled phase purity

Chia-Ying Su^a^, Chiu -Yen Chiu^b^, Jyh-Ming Ting^a,c,1^

a. Department of Materials Science and Engineering, National Cheng Kung University

Tainan, Taiwan

b. Material and Chemical Research, Industrial Technology Research Institute

Hsinchu, Taiwan

c. Research Center for Energy Technology and Strategy

National Cheng Kung University


S1. XRD pattern of Zn/Cu sample.

S2. XRD pattern of Sn/Cu sample.

(A)

(B)


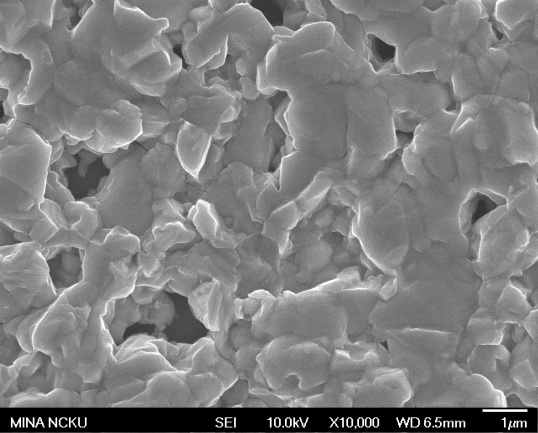
 (CZCT-1.8)

(C)


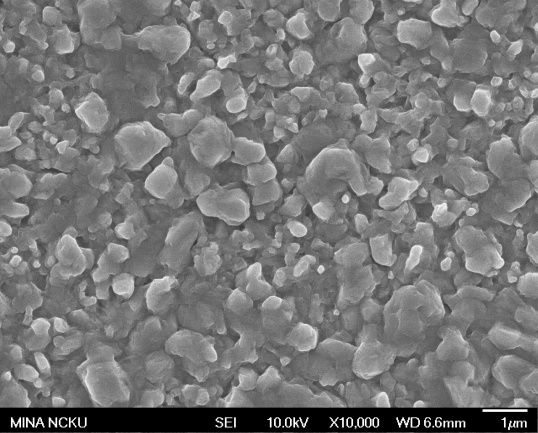
 (CZCT-0.9)

(D)

(E)

(F)

(G)

(H)

Fig. S3. Before the KCN treatment, (A) a sample with a higher Cu/(Sn+Zn) ratio shows Cu_2_S peaks but (B) the one with a lower ratio does not. Also, after KCN treatment, (C) a sample with a higher Cu/(Sn+Zn) ratio shows more surface removal of Cu_2_S, leaving pores on the surface, than (D) the one with a lower ratio. The other XRD patterns are shown in Figs using log scale: (E) CZCT-1.27 sample. (F) CZT group, (G) CTZ group and (H) CTZC group.
